# Supplementary material for: Association Studies on Gut and Lung Microbiomes in Patients with Lung Adenocarcinoma
Source: Microorganisms. 2023 Feb 21;11(3):546. doi: 10.3390/microorganisms11030546 (PMC10059697; doi:10.3390/microorganisms11030546)
Supplement: Supplementary file 1 [file microorganisms-11-00546-s001.zip › microorganisms-2150503-supplementary.pdf]

Supplemental Material

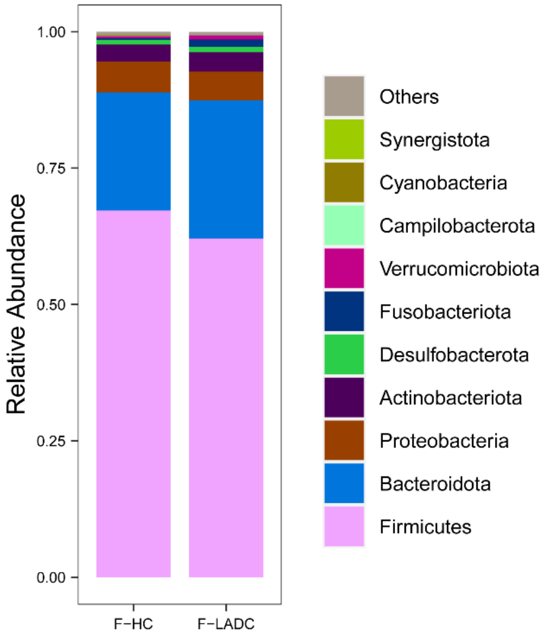

Figure S1. The composition of gut microbiota at the phylum level in LADC patients and healthy controls. Only the top 10 taxa are presented in the graph. F-HC, healthy control group; F-LADC, lung adenocarcinoma group.

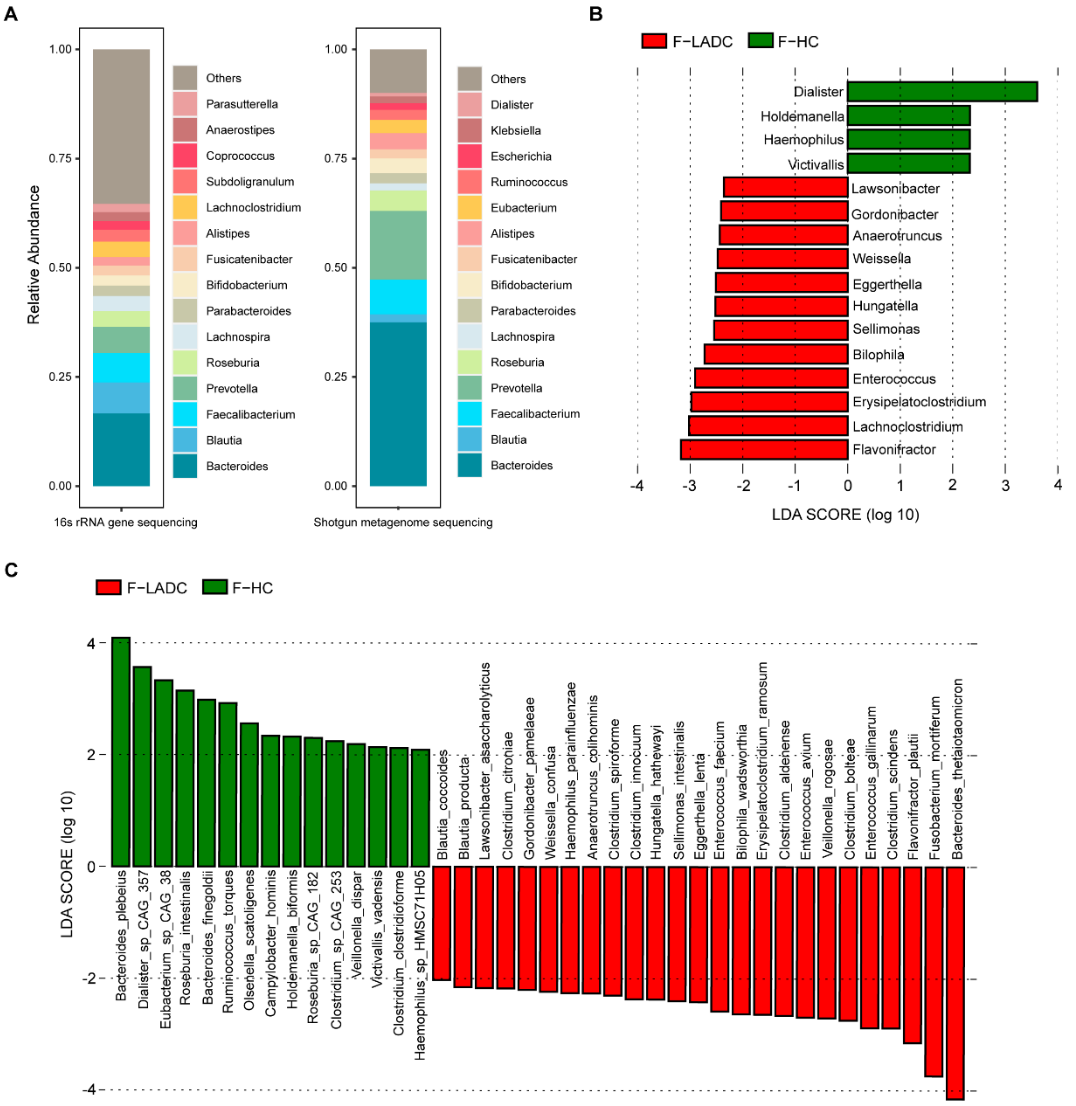

Figure S2. Differential abundance of gut microbiota in LADC and healthy controls using shotgun metagenome sequencing. (A) Comparison of the top 15 fecal microbiota at the genus level with 16S rRNA gene sequencing (left) and shotgun metagenome sequencing (right). Histogram of the LDA scores, where the LDA score indicates the effective size and ranking of taxa at the genus level (B) and the species level (C) (LDA > 2). Red and green represent lung adenocarcinoma patients (n = 43) and healthy controls (n = 64), respectively.

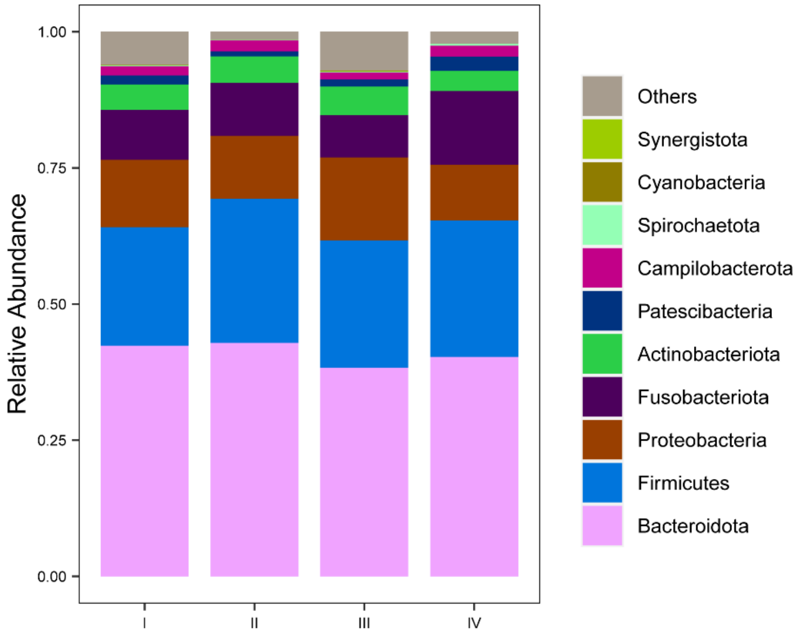

Figure S3. The composition of the lung microbiota at the phylum level in LADC. Only the top 10 phyla are presented in the graph. The bottom of the graph shows the disease staging status in LADC.

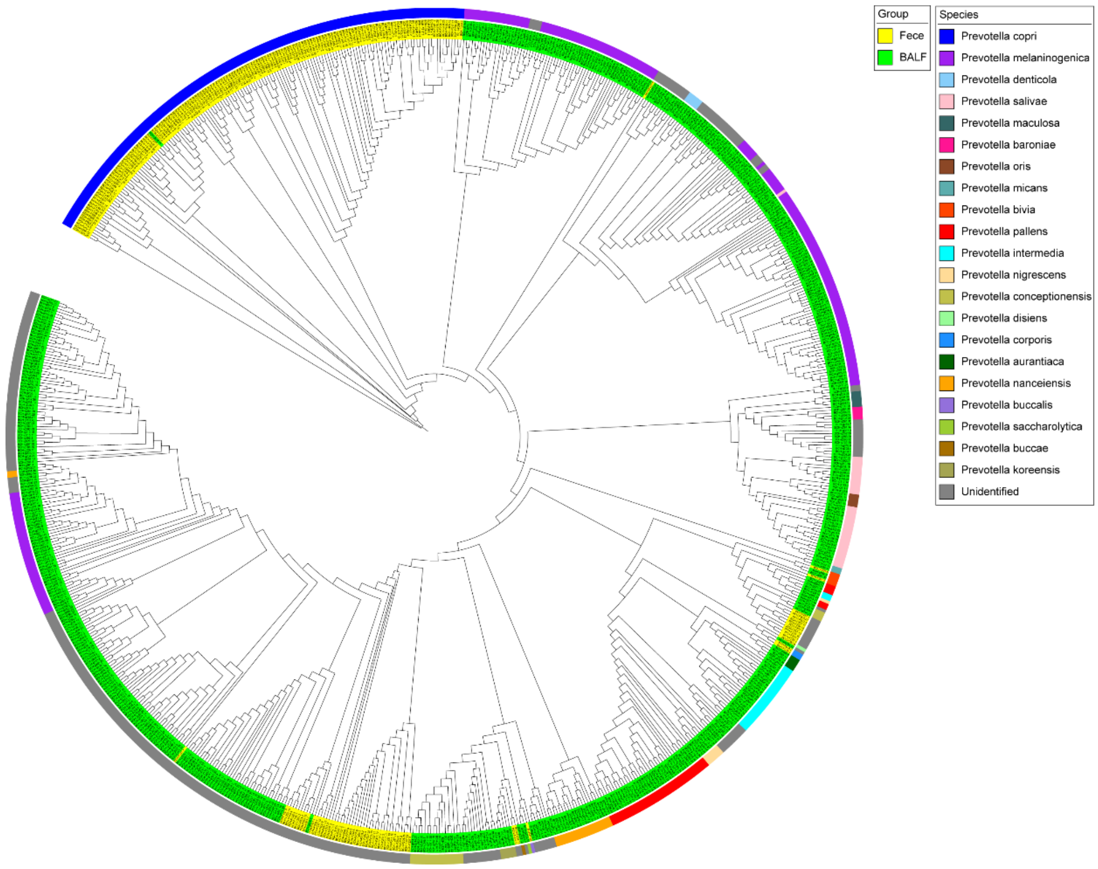

Figure S4. Phylogenetic analysis of the genus *Prevotella* with characteristic 16S rRNA gene sequences of paired fecal and BALF samples in patients with LADC. Yellow and green represent the sequences of fecal and BALF samples, respectively. The different colors in the outermost circle represent the annotated species. The phylogenetic analysis was conducted using FastTree software

Table S1. Counting the number of raw reads from 16S rRNA gene sequencing and shotgun metagenome sequencing

Sample ID	Group	Sample Type	Sequencing Type	The Number of Reads	The Number of Bases
BCH-F014	LADC	Feces	16S rRNA gene sequencing	208,272	104,136,000
BCH-F015	LADC	Feces	16S rRNA gene sequencing	182,633	91,316,500
BCH-F018	LADC	Feces	16S rRNA gene sequencing	83,266	41,633,000
BCH-F024	LADC	Feces	16S rRNA gene sequencing	170,240	85,120,000
BCH-F026	LADC	Feces	16S rRNA gene sequencing	59,401	29,700,500
BCH-F041	LADC	Feces	16S rRNA gene sequencing	155,336	77,668,000
BCH-F077	LADC	Feces	16S rRNA gene sequencing	234,504	117,252,000
BCH-F090	LADC	Feces	16S rRNA gene sequencing	145,239	72,619,500
BCH-F115	LADC	Feces	16S rRNA gene sequencing	188,581	94,290,500
BCH-F129	LADC	Feces	16S rRNA gene sequencing	82,542	41,271,000
BCH-F132	LADC	Feces	16S rRNA gene sequencing	296,955	148,477,500
BCH-F145	LADC	Feces	16S rRNA gene sequencing	213,082	106,541,000
BCH-F192	LADC	Feces	16S rRNA gene sequencing	191,847	95,923,500
BCH-F193	LADC	Feces	16S rRNA gene sequencing	197,466	98,733,000
BCH-F198	LADC	Feces	16S rRNA gene sequencing	181,064	90,532,000
BCH-F200	LADC	Feces	16S rRNA gene sequencing	177,409	88,704,500
BCH-F203	LADC	Feces	16S rRNA gene sequencing	169,710	84,855,000
BCH-F204	LADC	Feces	16S rRNA gene sequencing	211,642	105,821,000
BCH-F206	LADC	Feces	16S rRNA gene sequencing	197,229	98,614,500
BCH-F237	LADC	Feces	16S rRNA gene sequencing	210,423	105,211,500
BCH-F238	LADC	Feces	16S rRNA gene sequencing	278,100	139,050,000
BCH-F241	LADC	Feces	16S rRNA gene sequencing	171,339	85,669,500
BCH-F245	LADC	Feces	16S rRNA gene sequencing	173,543	86,771,500
BCH-F248	LADC	Feces	16S rRNA gene sequencing	267,155	133,577,500
BCH-F251	LADC	Feces	16S rRNA gene sequencing	166,760	83,380,000
BCH-F252	LADC	Feces	16S rRNA gene sequencing	212,609	106,304,500
BCH-F256	LADC	Feces	16S rRNA gene sequencing	141,619	70,809,500

BCH-F257	LADC	Feces	16S rRNA gene sequencing	135,578	67,789,000
BCH-F261	LADC	Feces	16S rRNA gene sequencing	238,178	119,089,000
BCH-F263	LADC	Feces	16S rRNA gene sequencing	129,494	64,747,000
BCH-F311	LADC	Feces	16S rRNA gene sequencing	189,562	94,781,000
BCH-F313	LADC	Feces	16S rRNA gene sequencing	102,798	51,399,000
BCH-F316	LADC	Feces	16S rRNA gene sequencing	193,998	96,999,000
BCH-F317	LADC	Feces	16S rRNA gene sequencing	189,282	94,641,000
BCH-F322	LADC	Feces	16S rRNA gene sequencing	223,462	111,731,000
BCH-F323	LADC	Feces	16S rRNA gene sequencing	205,792	102,896,000
BCH-F339	LADC	Feces	16S rRNA gene sequencing	126,230	63,115,000
BCH-F347	LADC	Feces	16S rRNA gene sequencing	178,957	89,478,500
BCH-F392	LADC	Feces	16S rRNA gene sequencing	197,833	98,916,500
BCH-F393	LADC	Feces	16S rRNA gene sequencing	134,012	67,006,000
BCH-F405	LADC	Feces	16S rRNA gene sequencing	108,684	54,342,000
BCH-F406	LADC	Feces	16S rRNA gene sequencing	132,424	66,212,000
BCH-F422	LADC	Feces	16S rRNA gene sequencing	227,792	113,896,000
BCH-g014	LADC	BALF	16S rRNA gene sequencing	140,020	70,010,000
BCH-g015	LADC	BALF	16S rRNA gene sequencing	114,464	57,232,000
BCH-g018	LADC	BALF	16S rRNA gene sequencing	174,760	87,380,000
BCH-g024	LADC	BALF	16S rRNA gene sequencing	217,604	108,802,000
BCH-g026	LADC	BALF	16S rRNA gene sequencing	249,683	124,841,500
BCH-g041	LADC	BALF	16S rRNA gene sequencing	139,929	69,964,500
BCH-g077	LADC	BALF	16S rRNA gene sequencing	138,397	69,198,500
BCH-g090	LADC	BALF	16S rRNA gene sequencing	191,881	95,940,500
BCH-g115	LADC	BALF	16S rRNA gene sequencing	179,343	89,671,500
BCH-g129	LADC	BALF	16S rRNA gene sequencing	146,795	73,397,500
BCH-g132	LADC	BALF	16S rRNA gene sequencing	104,368	52,184,000
BCH-g145	LADC	BALF	16S rRNA gene sequencing	171,815	85,907,500
BCH-g193	LADC	BALF	16S rRNA gene sequencing	128,456	64,228,000

BCH-g198	LADC	BALF	16S rRNA gene sequencing	149,237	74,618,500
BCH-g200	LADC	BALF	16S rRNA gene sequencing	160,276	80,138,000
BCH-g203	LADC	BALF	16S rRNA gene sequencing	154,296	77,148,000
BCH-g204	LADC	BALF	16S rRNA gene sequencing	162,335	81,167,500
BCH-g206	LADC	BALF	16S rRNA gene sequencing	139,703	69,851,500
BCH-g237	LADC	BALF	16S rRNA gene sequencing	114,330	57,165,000
BCH-g238	LADC	BALF	16S rRNA gene sequencing	147,032	73,516,000
BCH-g241	LADC	BALF	16S rRNA gene sequencing	127,942	63,971,000
BCH-g245	LADC	BALF	16S rRNA gene sequencing	122,406	61,203,000
BCH-g248	LADC	BALF	16S rRNA gene sequencing	200,548	100,274,000
BCH-g251	LADC	BALF	16S rRNA gene sequencing	112,723	56,361,500
BCH-g252	LADC	BALF	16S rRNA gene sequencing	146,509	73,254,500
BCH-g256	LADC	BALF	16S rRNA gene sequencing	130,378	65,189,000
BCH-g257	LADC	BALF	16S rRNA gene sequencing	184,076	92,038,000
BCH-g261	LADC	BALF	16S rRNA gene sequencing	118,524	59,262,000
BCH-g263	LADC	BALF	16S rRNA gene sequencing	127,792	63,896,000
BCH-g311	LADC	BALF	16S rRNA gene sequencing	165,175	82,587,500
BCH-g313	LADC	BALF	16S rRNA gene sequencing	163,583	81,791,500
BCH-g316	LADC	BALF	16S rRNA gene sequencing	104,785	52,392,500
BCH-g317	LADC	BALF	16S rRNA gene sequencing	188,349	94,174,500
BCH-g322	LADC	BALF	16S rRNA gene sequencing	104,036	52,018,000
BCH-g323	LADC	BALF	16S rRNA gene sequencing	181,943	90,971,500
BCH-g339	LADC	BALF	16S rRNA gene sequencing	46,143	23,071,500
BCH-g347	LADC	BALF	16S rRNA gene sequencing	53,624	26,812,000
BCH-g392	LADC	BALF	16S rRNA gene sequencing	148,775	74,387,500
BCH-g393	LADC	BALF	16S rRNA gene sequencing	121,750	60,875,000
BCH-g405	LADC	BALF	16S rRNA gene sequencing	130,233	65,116,500
BCH-g406	LADC	BALF	16S rRNA gene sequencing	176,657	88,328,500
BCH-g422	LADC	BALF	16S rRNA gene sequencing	180,936	90,468,000

BCH-h001	HC	Feces	16S rRNA gene sequencing	282,444	141,222,000
BCH-h002	HC	Feces	16S rRNA gene sequencing	111,882	55,941,000
BCH-h003	HC	Feces	16S rRNA gene sequencing	183,056	91,528,000
BCH-h004	HC	Feces	16S rRNA gene sequencing	171,812	85,906,000
BCH-h005	HC	Feces	16S rRNA gene sequencing	88,724	44,362,000
BCH-h006	HC	Feces	16S rRNA gene sequencing	64,169	32,084,500
BCH-h007	HC	Feces	16S rRNA gene sequencing	192,519	96,259,500
BCH-h008	HC	Feces	16S rRNA gene sequencing	222,833	111,416,500
BCH-h009	HC	Feces	16S rRNA gene sequencing	195,200	97,600,000
BCH-h010	HC	Feces	16S rRNA gene sequencing	182,105	91,052,500
BCH-h011	HC	Feces	16S rRNA gene sequencing	166,855	83,427,500
BCH-h012	HC	Feces	16S rRNA gene sequencing	89,399	44,699,500
BCH-h013	HC	Feces	16S rRNA gene sequencing	252,438	126,219,000
BCH-h014	HC	Feces	16S rRNA gene sequencing	201,148	100,574,000
BCH-h015	HC	Feces	16S rRNA gene sequencing	226,068	113,034,000
BCH-h016	HC	Feces	16S rRNA gene sequencing	207,711	103,855,500
BCH-h017	HC	Feces	16S rRNA gene sequencing	223,201	111,600,500
BCH-h018	HC	Feces	16S rRNA gene sequencing	250,230	125,115,000
BCH-h019	HC	Feces	16S rRNA gene sequencing	252,261	126,130,500
BCH-h020	HC	Feces	16S rRNA gene sequencing	246,864	123,432,000
BCH-h021	HC	Feces	16S rRNA gene sequencing	205,847	102,923,500
BCH-h022	HC	Feces	16S rRNA gene sequencing	179,816	89,908,000
BCH-h023	HC	Feces	16S rRNA gene sequencing	221,540	110,770,000
BCH-h024	HC	Feces	16S rRNA gene sequencing	298,021	149,010,500
BCH-h025	HC	Feces	16S rRNA gene sequencing	224,155	112,077,500
BCH-h026	HC	Feces	16S rRNA gene sequencing	204,573	102,286,500
BCH-h027	HC	Feces	16S rRNA gene sequencing	186,258	93,129,000
BCH-h028	HC	Feces	16S rRNA gene sequencing	209,405	104,702,500
BCH-h029	HC	Feces	16S rRNA gene sequencing	213,064	106,532,000

BCH-h030	HC	Feces	16S rRNA gene sequencing	215,017	107,508,500
BCH-h031	HC	Feces	16S rRNA gene sequencing	184,986	92,493,000
BCH-h032	HC	Feces	16S rRNA gene sequencing	218,963	109,481,500
BCH-h033	HC	Feces	16S rRNA gene sequencing	236,738	118,369,000
BCH-h034	HC	Feces	16S rRNA gene sequencing	197,837	98,918,500
BCH-h035	HC	Feces	16S rRNA gene sequencing	212,444	106,222,000
BCH-h036	HC	Feces	16S rRNA gene sequencing	228,024	114,012,000
BCH-h037	HC	Feces	16S rRNA gene sequencing	171,942	85,971,000
BCH-h038	HC	Feces	16S rRNA gene sequencing	232,247	116,123,500
BCH-h039	HC	Feces	16S rRNA gene sequencing	189,156	94,578,000
BCH-h040	HC	Feces	16S rRNA gene sequencing	196,047	98,023,500
BCH-h041	HC	Feces	16S rRNA gene sequencing	258,652	129,326,000
BCH-h042	HC	Feces	16S rRNA gene sequencing	254,972	127,486,000
BCH-h043	HC	Feces	16S rRNA gene sequencing	268,280	134,140,000
BCH-h044	HC	Feces	16S rRNA gene sequencing	199,079	99,539,500
BCH-h045	HC	Feces	16S rRNA gene sequencing	152,252	76,126,000
BCH-h046	HC	Feces	16S rRNA gene sequencing	157,262	78,631,000
BCH-h047	HC	Feces	16S rRNA gene sequencing	249,791	124,895,500
BCH-h048	HC	Feces	16S rRNA gene sequencing	218,213	109,106,500
BCH-h049	HC	Feces	16S rRNA gene sequencing	238,754	119,377,000
BCH-h050	HC	Feces	16S rRNA gene sequencing	200,203	100,101,500
BCH-h051	HC	Feces	16S rRNA gene sequencing	237,356	118,678,000
BCH-h052	HC	Feces	16S rRNA gene sequencing	139,922	69,961,000
BCH-h053	HC	Feces	16S rRNA gene sequencing	241,615	120,807,500
BCH-h054	HC	Feces	16S rRNA gene sequencing	163,334	81,667,000
BCH-h055	HC	Feces	16S rRNA gene sequencing	163,628	81,814,000
BCH-h056	HC	Feces	16S rRNA gene sequencing	180,584	90,292,000
BCH-h057	HC	Feces	16S rRNA gene sequencing	214,024	107,012,000
BCH-h058	HC	Feces	16S rRNA gene sequencing	218,133	109,066,500

BCH-h059	HC	Feces	16S rRNA gene sequencing	297,999	148,999,500
BCH-h060	HC	Feces	16S rRNA gene sequencing	246,281	123,140,500
BCH-h061	HC	Feces	16S rRNA gene sequencing	218,590	109,295,000
BCH-h062	HC	Feces	16S rRNA gene sequencing	236,382	118,191,000
BCH-h063	HC	Feces	16S rRNA gene sequencing	228,405	114,202,500
BCH-h064	HC	Feces	16S rRNA gene sequencing	238,064	119,032,000
BCH-F014	LADC	Feces	shotgun metagenome sequencing	83,579,256	12,536,888,400
BCH-F015	LADC	Feces	shotgun metagenome sequencing	60,438,564	9,065,784,600
BCH-F018	LADC	Feces	shotgun metagenome sequencing	54,189,254	8,128,388,100
BCH-F024	LADC	Feces	shotgun metagenome sequencing	60,489,316	9,073,397,400
BCH-F026	LADC	Feces	shotgun metagenome sequencing	63,621,804	9,543,270,600
BCH-F041	LADC	Feces	shotgun metagenome sequencing	70,182,530	10,527,379,500
BCH-F077	LADC	Feces	shotgun metagenome sequencing	71,065,372	10,659,805,800
BCH-F090	LADC	Feces	shotgun metagenome sequencing	71,066,812	10,660,021,800
BCH-F115	LADC	Feces	shotgun metagenome sequencing	101,034,756	15,155,213,400
BCH-F129	LADC	Feces	shotgun metagenome sequencing	87,146,454	13,071,968,100
BCH-F132	LADC	Feces	shotgun metagenome sequencing	76,781,440	11,517,216,000
BCH-F145	LADC	Feces	shotgun metagenome sequencing	94,763,308	14,214,496,200
BCH-F192	LADC	Feces	shotgun metagenome sequencing	79,714,602	11,957,190,300
BCH-F193	LADC	Feces	shotgun metagenome sequencing	84,389,134	12,658,370,100
BCH-F198	LADC	Feces	shotgun metagenome sequencing	80,436,758	12,065,513,700
BCH-F200	LADC	Feces	shotgun metagenome sequencing	76,290,550	11,443,582,500
BCH-F203	LADC	Feces	shotgun metagenome sequencing	78,513,346	11,777,001,900
BCH-F204	LADC	Feces	shotgun metagenome sequencing	85,889,730	12,883,459,500
BCH-F206	LADC	Feces	shotgun metagenome sequencing	85,733,452	12,860,017,800
BCH-F237	LADC	Feces	shotgun metagenome sequencing	75,403,122	11,310,468,300
BCH-F238	LADC	Feces	shotgun metagenome sequencing	74,511,096	11,176,664,400
BCH-F241	LADC	Feces	shotgun metagenome sequencing	60,479,170	9,071,875,500
BCH-F245	LADC	Feces	shotgun metagenome sequencing	71,110,442	10,666,566,300

BCH-F248	LADC	Feces	shotgun metagenome sequencing	96,975,396	14,546,309,400
BCH-F251	LADC	Feces	shotgun metagenome sequencing	97,210,826	14,581,623,900
BCH-F252	LADC	Feces	shotgun metagenome sequencing	103,803,122	15,570,468,300
BCH-F256	LADC	Feces	shotgun metagenome sequencing	85,725,074	12,858,761,100
BCH-F257	LADC	Feces	shotgun metagenome sequencing	67,154,046	10,073,106,900
BCH-F261	LADC	Feces	shotgun metagenome sequencing	84,378,070	12,656,710,500
BCH-F263	LADC	Feces	shotgun metagenome sequencing	88,751,788	13,312,768,200
BCH-F311	LADC	Feces	shotgun metagenome sequencing	78,814,038	11,822,105,700
BCH-F313	LADC	Feces	shotgun metagenome sequencing	69,894,904	10,484,235,600
BCH-F316	LADC	Feces	shotgun metagenome sequencing	87,075,528	13,061,329,200
BCH-F317	LADC	Feces	shotgun metagenome sequencing	74,826,044	11,223,906,600
BCH-F322	LADC	Feces	shotgun metagenome sequencing	68,788,360	10,318,254,000
BCH-F323	LADC	Feces	shotgun metagenome sequencing	55,235,254	8,285,288,100
BCH-F339	LADC	Feces	shotgun metagenome sequencing	68,878,874	10,331,831,100
BCH-F347	LADC	Feces	shotgun metagenome sequencing	66,792,630	10,018,894,500
BCH-F392	LADC	Feces	shotgun metagenome sequencing	55,189,148	8,278,372,200
BCH-F393	LADC	Feces	shotgun metagenome sequencing	54,852,382	8,227,857,300
BCH-F405	LADC	Feces	shotgun metagenome sequencing	63,086,430	9,462,964,500
BCH-F406	LADC	Feces	shotgun metagenome sequencing	62,392,382	9,358,857,300
BCH-F422	LADC	Feces	shotgun metagenome sequencing	66,176,464	9,926,469,600
BCH-h001	HC	Feces	shotgun metagenome sequencing	95,857,824	14,378,673,600
BCH-h002	HC	Feces	shotgun metagenome sequencing	82,983,508	12,447,526,200
BCH-h003	HC	Feces	shotgun metagenome sequencing	80,886,752	12,133,012,800
BCH-h004	HC	Feces	shotgun metagenome sequencing	86,735,000	13,010,250,000
BCH-h005	HC	Feces	shotgun metagenome sequencing	83,390,086	12,508,512,900
BCH-h006	HC	Feces	shotgun metagenome sequencing	93,686,228	14,052,934,200
BCH-h007	HC	Feces	shotgun metagenome sequencing	95,790,832	14,368,624,800
BCH-h008	HC	Feces	shotgun metagenome sequencing	56,955,848	8,543,377,200
BCH-h009	HC	Feces	shotgun metagenome sequencing	54,759,016	8,213,852,400

BCH-h010	HC	Feces	shotgun metagenome sequencing	62,587,136	9,388,070,400
BCH-h011	HC	Feces	shotgun metagenome sequencing	88,540,140	13,281,021,000
BCH-h012	HC	Feces	shotgun metagenome sequencing	90,047,538	13,507,130,700
BCH-h013	HC	Feces	shotgun metagenome sequencing	89,153,542	13,373,031,300
BCH-h014	HC	Feces	shotgun metagenome sequencing	53,780,394	8,067,059,100
BCH-h015	HC	Feces	shotgun metagenome sequencing	88,366,448	13,254,967,200
BCH-h016	HC	Feces	shotgun metagenome sequencing	87,928,342	13,189,251,300
BCH-h017	HC	Feces	shotgun metagenome sequencing	71,671,238	10,750,685,700
BCH-h018	HC	Feces	shotgun metagenome sequencing	76,840,404	11,526,060,600
BCH-h019	HC	Feces	shotgun metagenome sequencing	52,735,286	7,910,292,900
BCH-h020	HC	Feces	shotgun metagenome sequencing	50,045,596	7,506,839,400
BCH-h021	HC	Feces	shotgun metagenome sequencing	74,345,026	11,151,753,900
BCH-h022	HC	Feces	shotgun metagenome sequencing	53,779,520	8,066,928,000
BCH-h023	HC	Feces	shotgun metagenome sequencing	73,117,280	10,967,592,000
BCH-h024	HC	Feces	shotgun metagenome sequencing	62,565,090	9,384,763,500
BCH-h025	HC	Feces	shotgun metagenome sequencing	70,449,384	10,567,407,600
BCH-h026	HC	Feces	shotgun metagenome sequencing	64,872,584	9,730,887,600
BCH-h027	HC	Feces	shotgun metagenome sequencing	67,967,318	10,195,097,700
BCH-h028	HC	Feces	shotgun metagenome sequencing	82,562,732	12,384,409,800
BCH-h029	HC	Feces	shotgun metagenome sequencing	99,440,806	14,916,120,900
BCH-h030	HC	Feces	shotgun metagenome sequencing	75,339,692	11,300,953,800
BCH-h031	HC	Feces	shotgun metagenome sequencing	69,396,684	10,409,502,600
BCH-h032	HC	Feces	shotgun metagenome sequencing	80,018,574	12,002,786,100
BCH-h033	HC	Feces	shotgun metagenome sequencing	61,853,524	9,278,028,600
BCH-h034	HC	Feces	shotgun metagenome sequencing	54,314,628	8,147,194,200
BCH-h035	HC	Feces	shotgun metagenome sequencing	83,747,436	12,562,115,400
BCH-h036	HC	Feces	shotgun metagenome sequencing	87,393,110	13,108,966,500
BCH-h037	HC	Feces	shotgun metagenome sequencing	56,261,328	8,439,199,200
BCH-h038	HC	Feces	shotgun metagenome sequencing	78,279,886	11,741,982,900

BCH-h039	HC	Feces	shotgun metagenome sequencing	70,526,676	10,579,001,400
BCH-h040	HC	Feces	shotgun metagenome sequencing	55,282,294	8,292,344,100
BCH-h041	HC	Feces	shotgun metagenome sequencing	76,536,588	11,480,488,200
BCH-h042	HC	Feces	shotgun metagenome sequencing	74,785,776	11,217,866,400
BCH-h043	HC	Feces	shotgun metagenome sequencing	75,054,358	11,258,153,700
BCH-h044	HC	Feces	shotgun metagenome sequencing	72,840,558	10,926,083,700
BCH-h045	HC	Feces	shotgun metagenome sequencing	87,101,812	13,065,271,800
BCH-h046	HC	Feces	shotgun metagenome sequencing	93,060,652	13,959,097,800
BCH-h047	HC	Feces	shotgun metagenome sequencing	42,315,778	6,347,366,700
BCH-h048	HC	Feces	shotgun metagenome sequencing	74,915,688	11,237,353,200
BCH-h049	HC	Feces	shotgun metagenome sequencing	90,302,024	13,545,303,600
BCH-h050	HC	Feces	shotgun metagenome sequencing	69,184,202	10,377,630,300
BCH-h051	HC	Feces	shotgun metagenome sequencing	55,859,676	8,378,951,400
BCH-h052	HC	Feces	shotgun metagenome sequencing	79,725,436	11,958,815,400
BCH-h053	HC	Feces	shotgun metagenome sequencing	62,625,902	9,393,885,300
BCH-h054	HC	Feces	shotgun metagenome sequencing	69,650,230	10,447,534,500
BCH-h055	HC	Feces	shotgun metagenome sequencing	83,605,336	12,540,800,400
BCH-h056	HC	Feces	shotgun metagenome sequencing	81,957,160	12,293,574,000
BCH-h057	HC	Feces	shotgun metagenome sequencing	79,094,104	11,864,115,600
BCH-h058	HC	Feces	shotgun metagenome sequencing	71,254,844	10,688,226,600
BCH-h059	HC	Feces	shotgun metagenome sequencing	28,547,858	4,282,178,700
BCH-h060	HC	Feces	shotgun metagenome sequencing	79,286,802	11,893,020,300
BCH-h061	HC	Feces	shotgun metagenome sequencing	86,228,650	12,934,297,500
BCH-h062	HC	Feces	shotgun metagenome sequencing	71,356,912	10,703,536,800
BCH-h063	HC	Feces	shotgun metagenome sequencing	84,524,720	12,678,708,000
BCH-h064	HC	Feces	shotgun metagenome sequencing	69,557,306	10,433,595,900

Table S2. Counting the number of shared genera both in paired fecal samples and BALF samples from 42 LADC patients

Shared genera	No. of patients	The relative abundance in gut	The relative abundance in lung
Streptococcus	26	0.007947846	0.028945578
Prevotella	23	0.050124717	0.191882086
Fusobacterium	13	0.013798186	0.048185941
Haemophilus	10	0.002891156	0.032823129
Bacteroides	10	0.149580499	0.004863946
Bifidobacterium	9	0.020555556	0.002301587
Dialister	8	0.005362812	0.003310658
Faecalibacterium	7	0.057165533	0.002947846
Actinomyces	6	0.000385487	0.023786848
Veillonella	5	0.001587302	0.082562358
Rothia	5	0.000181406	0.011984127
Megasphaera	5	0.009229025	0.01244898
Peptococcus	3	0.000238095	0.001088435
Neisseria	3	0.000170068	0.050907029
Klebsiella	3	0.003049887	0.003639456
Collinsella	3	0.008015873	0.00069161
Blautia	3	0.058764172	0.000612245

Only shared bacteria present in more than 3 (inclusive) patients are shown in the table. The relative abundance shows the average.

Table S3. Statistics of shared species of the genus *Streptococcus*

Sample id	No. of sequences from gut	No. of sequences from lung	Shared species	Annotation species (No. of sequences from gut)	Annotation species (No. of sequences from lung)
BCH-015	1	3	----	*Streptococcus parasanguinis* (1)	*Streptococcus salivarius* (1)
BCH-024	2	4	*Streptococcus salivarius*	*Streptococcus salivarius* (1)	*Streptococcus salivarius* (1); *Streptococcus parasanguinis* (1); *Streptococcus sanguinis* (1)
BCH-026	1	9	*Streptococcus salivarius*	*Streptococcus salivarius* (1)	*Streptococcus salivarius* (2); *Streptococcus oralis* (1); *Streptococcus cristatus* (1); *Streptococcus parasanguinis* (1)
BCH-041	3	2	----	*Streptococcus parasanguinis* (1); *Streptococcus salivarius* (1)	Unidentified
BCH-090	2	5	----	*Streptococcus salivarius* (1)	*Streptococcus anginosus* (1)
BCH-115	1	2	----	*Streptococcus cristatus* (1)	*Streptococcus anginosus* (1)
BCH-145	1	3	----	*Streptococcus gordonii* (1)	*Streptococcus cristatus* (1)
BCH-193	1	2	----	Unidentified	*Streptococcus cristatus* (1)
BCH-203	1	4	----	Unidentified	*Streptococcus anginosus* (1); *Streptococcus parasanguinis* (1)
BCH-206	3	1	----	*Streptococcus salivarius* (1)	*Streptococcus salivarius* (1)
BCH-238	2	1	----	*Streptococcus gallolyticus* (1)	*Streptococcus cristatus* (1)
BCH-241	1	1	----	Unidentified	Unidentified
BCH-245	2	2	*Streptococcus salivarius*	*Streptococcus salivarius* (2)	*Streptococcus salivarius* (1); *Streptococcus parasanguinis* (1)
BCH-248	2	2	----	*Streptococcus cristatus* (1)	Unidentified
BCH-251	1	1	----	*Streptococcus parasanguinis* (1)	Unidentified
BCH-252	1	3	----	Unidentified	*Streptococcus peroris* (1)
BCH-311	2	8	----	*Streptococcus gallolyticus* (1)	*Streptococcus cristatus* (1)
BCH-313	4	12	----	*Streptococcus salivarius* (3)	*Streptococcus cristatus* (1)
BCH-316	1	5	----	Unidentified	*Streptococcus salivarius* (1); *Streptococcus oralis* (1); *Streptococcus parasanguinis* (1)
BCH-323	1	6	----	*Streptococcus salivarius* (1)	*Streptococcus cristatus* (1)
BCH-339	1	1	----	Unidentified	Unidentified
BCH-347	1	1	----	*Streptococcus salivarius* (1)	Unidentified
BCH-393	1	4	*Streptococcus salivarius*	*Streptococcus salivarius* (1)	*Streptococcus salivarius* (2); *Streptococcus pneumoniae* (1);

					Streptococcus cristatus (1)
BCH-405	1	4	----	*Streptococcus salivarius* (1)	*Streptococcus parasanguinis* (1); *Streptococcus oralis* (1)
BCH-406	2	14	*Streptococcus salivarius*	*Streptococcus salivarius* (1)	*Streptococcus salivarius* (1); *Streptococcus parasanguinis* (1); *Streptococcus oralis* (1)
BCH-422	2	3	----	*Streptococcus gallolyticus* (1); *Streptococcus salivarius* (1)	Unidentified

The *Streptococcus* had 144 16S rRNA gene sequences in 26 patients, among which 41 sequences from gut samples and 103 sequences from lung samples. We annotated 10 species, of which gut samples were annotated to 5 species and BALF samples to 8 species. The result identified the share species was *Streptococcus salivarius* in 5 patients. The sequences from paired fecal and BALF samples in LADC patients with distant phylogenetic distance and low sequence identity (< 97%) were not considered as shared species. The sequence identity of pairwise 16S rRNA gene sequences was analyzed using VSEARCH v2.15.0.

Table S4. Statistics of shared species of the genus *Fusobacterium*

Sample id	No. of sequences from gut	No. of sequences from lung	Shared species	Annotation species (No. of sequences from gut)	Annotation species (No. of sequences from lung)
BCH-024	1	1	----	*Fusobacterium mortiferum* (1)	*Fusobacterium periodonticum* (1)
BCH-026	2	8	----	*Fusobacterium mortiferum* (1)	*Fusobacterium mortiferum* (1); *Fusobacterium nucleatum* (3); *Fusobacterium periodonticum* (4)
BCH-041	18	8	----	*Fusobacterium mortiferum* (18)	*Fusobacterium nucleatum* (1); *Fusobacterium periodonticum* (7)
BCH-090	1	2	----	*Fusobacterium ulcerans* (1)	*Fusobacterium nucleatum* (2)
BCH-132	1	1	----	*Fusobacterium mortiferum* (1)	*Fusobacterium periodonticum* (1)
BCH-145	1	5	----	*Fusobacterium mortiferum* (1)	*Fusobacterium nucleatum* (2); *Fusobacterium periodonticum* (3)
BCH-203	1	1	----	*Fusobacterium mortiferum* (1)	*Fusobacterium nucleatum* (1)
BCH-238	30	5	*Fusobacterium mortiferum*	*Fusobacterium mortiferum* (26)	*Fusobacterium mortiferum* (1); *Fusobacterium periodonticum* (4)
BCH-241	3	9	----	*Fusobacterium mortiferum* (1); *Fusobacterium ulcerans* (1)	*Fusobacterium nucleatum* (4); *Fusobacterium periodonticum* (5)
BCH-251	1	4	----	*Fusobacterium mortiferum* (1)	*Fusobacterium nucleatum* (4)
BCH-256	34	4	----	*Fusobacterium mortiferum* (29)	*Fusobacterium periodonticum* (4)
BCH-311	1	4	----	*Fusobacterium mortiferum* (1)	*Fusobacterium nucleatum* (2); *Fusobacterium periodonticum* (2)
BCH-322	1	1	----	*Fusobacterium mortiferum* (1)	*Fusobacterium periodonticum* (1)

The *Fusobacterium* had 148 16S rRNA gene sequences in 13 patients, among which 95 sequences from gut samples and 53 sequences from lung samples. We annotated 5 species, of which gut samples were annotated to 2 species and BALF samples to 4 species. The result identified the share species was *Fusobacterium mortiferum* in 1 patient. The sequences from paired fecal and BALF samples in LADC patients with distant phylogenetic distance and low sequence identity (< 97%) were not considered as shared species. The sequence identity of pairwise 16S rRNA gene sequences was analyzed using VSEARCH v2.15.0.

Table S5. Statistics of shared species of the genus *Haemophilus*

Sample id	No. of sequences from gut	No. of sequences from lung	Shared species	Annotation species (No. of sequences from gut)	Annotation species (No. of sequences from lung)
BCH-026	1	1	*Haemophilus parainfluenzae*	*Haemophilus parainfluenzae* (1)	*Haemophilus parainfluenzae* (1)
BCH-115	1	1	*Haemophilus parainfluenzae*	*Haemophilus parainfluenzae* (1)	*Haemophilus parainfluenzae* (1)
BCH-145	1	2	*Haemophilus parainfluenzae*	*Haemophilus parainfluenzae* (1)	*Haemophilus parainfluenzae* (1); *Haemophilus influenzae* (1)
BCH-193	1	1	*Haemophilus parainfluenzae*	*Haemophilus parainfluenzae* (1)	*Haemophilus parainfluenzae* (1)
BCH-198	1	1	*Haemophilus parainfluenzae*	*Haemophilus parainfluenzae* (1)	*Haemophilus parainfluenzae* (1)
BCH-200	6	1	*Haemophilus parainfluenzae*	*Haemophilus parainfluenzae* (6)	*Haemophilus parainfluenzae* (1)
BCH-203	1	1	*Haemophilus parainfluenzae*	*Haemophilus parainfluenzae* (1)	*Haemophilus parainfluenzae* (1)
BCH-206	4	3	*Haemophilus parainfluenzae*	*Haemophilus parainfluenzae* (4)	*Haemophilus parainfluenzae* (2)
BCH-241	1	1	*Haemophilus parainfluenzae*	*Haemophilus parainfluenzae* (1)	*Haemophilus parainfluenzae* (1)
BCH-313	1	2	*Haemophilus parainfluenzae*	*Haemophilus parainfluenzae* (1)	*Haemophilus parainfluenzae* (1); *Haemophilus parahaemolyticus* (1)

The *Haemophilus* had 32 16S rRNA gene sequences in 10 patients, among which 18 sequences from gut samples and 14 sequences from lung samples. We annotated 3 species, of which gut samples were annotated to 1 species and BALF samples to 3 species. The result identified the share species was *Haemophilus parainfluenzae* in 10 patients. The sequences from paired fecal and BALF samples in LADC patients with distant phylogenetic distance and low sequence identity (< 97%) were not considered as shared species. The sequence identity of pairwise 16S rRNA gene sequences was analyzed using VSEARCH v2.15.0.

Table S6. Statistics of shared species of the genus *Bacteroides*

Sample id	No. of sequences from gut	No. of sequences from lung	Shared species	Annotation species (No. of sequences from gut)	Annotation species (No. of sequences from lung)
BCH-015	33	1	----	*Bacteroides finegoldii* (1); *Bacteroides intestinalis* (1); *Bacteroides ovatus* (1); *Bacteroides thetaiotaomicron* (3); *Bacteroides uniformis* (5); *Bacteroides xylanisolvens* (1)	Unidentified
BCH-018	13	8	----	*Bacteroides ovatus* (2); *Bacteroides stercoris* (1)	*Bacteroides uniformis* (2); *Bacteroides stercoris* (1)
BCH-024	18	3	----	*Bacteroides thetaiotaomicron* (12); *Bacteroides fragilis* (2)	Unidentified
BCH-090	37	3	*Bacteroides fragilis*	*Bacteroides caccae* (1); *Bacteroides eggerthii* (2); *Bacteroides finegoldii* (1); *Bacteroides fragilis* (2); *Bacteroides nordii* (1); *Bacteroides ovatus* (3); *Bacteroides stercoris* (6); *Bacteroides thetaiotaomicron* (1); *Bacteroides uniformis* (5); *Bacteroides xylanisolvens* (2)	*Bacteroides fragilis* (2)
BCH-115	33	1	----	*Bacteroides finegoldii* (1); *Bacteroides ovatus* (2); *Bacteroides thetaiotaomicron* (2); *Bacteroides uniformis* (1); *Bacteroides xylanisolvens* (4)	*Bacteroides stercoris* (1)
BCH-132	42	1	----	*Bacteroides ovatus* (3); *Bacteroides stercoris* (6); *Bacteroides thetaiotaomicron* (2); *Bacteroides uniformis* (1)	Unidentified
BCH-203	27	3	----	*Bacteroides caccae* (1); *Bacteroides ovatus* (2); *Bacteroides stercoris* (2); *Bacteroides xylanisolvens* (1); *Bacteroides thetaiotaomicron* (1)	*Bacteroides fragilis* (1)

BCH-256	29	11	*Bacteroides uniformis*	*Bacteroides caccae* (1); *Bacteroides cellulosilyticus* (1); *Bacteroides nordii* (1); *Bacteroides stercoris* (2); *Bacteroides thetaiotaomicron* (1); *Bacteroides uniformis* (1)	*Bacteroides uniformis* (1); *Bacteroides intestinal* (3)
BCH-393	54	7	*Bacteroides stercoris*	*Bacteroides stercoris* (7); *Bacteroides cellulosilyticus* (1); *Bacteroides caccae* (1); *Bacteroides finegoldii* (1); *Bacteroides thetaiotaomicron* (1); *Bacteroides uniformis* (2); *Bacteroides xylanisolvens* (1)	*Bacteroides stercoris* (1); *Bacteroides ovatus* (1); *Bacteroides uniformis* (1)
BCH-422	66	1	----	*Bacteroides uniformis* (7); *Bacteroides xylanisolvens* (6); *Bacteroides eggerthii* (3); *Bacteroides ovatus* (4); *Bacteroides finegoldii* (1); *Bacteroides stercoris* (1)	Unidentified

The *Bacteroides* had 391 16S rRNA gene sequences in 10 patients, among which 352 sequences from gut samples and 39 sequences from lung samples. We annotated 12 species, of which gut samples were annotated to 12 species and BALF samples to 5 species. The result identified the share species were *Bacteroides fragilis* in 1 patient, *Bacteroides uniformis* in 1 patient and *Bacteroides stercoris* in 1 patient. The sequences from paired fecal and BALF samples in LADC patients with distant phylogenetic distance and low sequence identity (< 97%) were not considered as shared species. The sequence identity of pairwise 16S rRNA gene sequences was analyzed using VSEARCH v2.15.0.

Table S7. Statistics of shared species of the genus *Prevotella*

Sample id	No. of sequences from gut	No. of sequences from lung	Shared species	Annotation species (No. of sequences from gut)	Annotation species (No. of sequences from lung)
BCH-014	5	29	----	*Prevotella stercorea* (1)	*Prevotella shahii* (3); *Prevotella melaninogenica* (3); *Prevotella intermedia* (2); *Prevotella nanceiensis* (2); *Prevotella salivae* (1); *Prevotella pallens* (1)
BCH-015	1	12	----	*Prevotella copri* (1)	*Prevotella shahii* (1); *Prevotella melaninogenica* (2); *Prevotella nanceiensis* (1); *Prevotella salivae* (1); *Prevotella pallens* (1)
BCH-018	2	13	----	*Prevotella corporis* (1)	*Prevotella melaninogenica* (2); *Prevotella salivae* (1); *Prevotella pallens* (1); *Prevotella koreensis* (1)
BCH-024	3	5	----	*Prevotella copri* (1)	*Prevotella melaninogenica* (2); *Prevotella salivae* (1); *Prevotella jejuni* (1)
BCH-026	8	26	----	*Prevotella copri* (5)	*Prevotella melaninogenica* (6); *Prevotella salivae* (3); *Prevotella pallens* (4); *Prevotella nanceiensis* (1); *Prevotella oris* (1); *Prevotella intermedia* (1)
BCH-077	1	30	----	*Prevotella copri* (1)	*Prevotella melaninogenica* (7); *Prevotella pallens* (1); *Prevotella nanceiensis* (2); *Prevotella intermedia* (1)
BCH-090	1	32	----	*Prevotella copri* (1)	*Prevotella nigrescens* (1); *Prevotella jejuni* (1); *Prevotella oris* (1); *Prevotella intermedia* (2); *Prevotella melaninogenica* (1); *Prevotella nanceiensis* (2); *Prevotella denticola* (2); *Prevotella oulorum* (1); *Prevotella multiformis* (1)
BCH-132	3	23	----	*Prevotella copri* (1)	*Prevotella shahii* (1); *Prevotella micans* (1); *Prevotella melaninogenica* (3); *Prevotella koreensis* (1); *Prevotella intermedia* (1); *Prevotella pallens* (1); *Prevotella salivae* (1)

BCH-193	1	46	----	*Prevotella buccalis* (1)	*Prevotella melaninogenica* (8); *Prevotella nanceiensis* (1); *Prevotella salivae* (2); *Prevotella denticola* (1); *Prevotella veroralis* (1); *Prevotella pallens* (2); *Prevotella multiformis* (1); *Prevotella baroniae* (1); *Prevotella shahii* (2); *Prevotella oris* (1)
BCH-203	2	16	----	*Prevotella copri* (1); *Prevotella stercorea* (1)	*Prevotella melaninogenica* (4); *Prevotella intermedia* (2); *Prevotella salivae* (1); *Prevotella pallens* (1); *Prevotella pleuritidis* (1); *Prevotella nanceiensis* (1); *Prevotella fusca* (1)
BCH-204	25	41	----	*Prevotella copri* (7); *Prevotella stercorea* (2)	*Prevotella melaninogenica* (5); *Prevotella intermedia* (2); *Prevotella baroniae* (1); *Prevotella pallens* (1); *Prevotella maculosa* (1); *Prevotella salivae* (2); *Prevotella shahii* (1); *Prevotella jejuni* (1)
BCH-237	25	38	----	*Prevotella copri* (12); *Prevotella stercorea* (6)	*Prevotella melaninogenica* (11); *Prevotella scopos* (1); *Prevotella histicola* (1); *Prevotella salivae* (1); *Prevotella pallens* (1)
BCH-248	30	25	----	*Prevotella copri* (23)	*Prevotella melaninogenica* (5); *Prevotella nanceiensis* (2); *Prevotella salivae* (3); *Prevotella pallens* (1); *Prevotella shahii* (1); *Prevotella aurantiaca* (1)
BCH-252	1	30	----	*Prevotella copri* (1)	*Prevotella intermedia* (4); *Prevotella koreensis* (1); *Prevotella saccharolytica* (1); *Prevotella nanceiensis* (1); *Prevotella shahii* (1); *Prevotella pallens* (1); *Prevotella aurantiaca* (1); *Prevotella melaninogenica* (1)
BCH-256	14	15	----	*Prevotella copri* (10)	*Prevotella melaninogenica* (4); *Prevotella salivae* (1); *Prevotella shahii* (1); *Prevotella nanceiensis* (1); *Prevotella pallens* (1)

BCH-257	15	38	----	*Prevotella stercorea* (10)	*Prevotella melaninogenica* (9); *Prevotella oris* (1); *Prevotella buccae* (1); *Prevotella salivae* (3); *Prevotella pallens* (1); *Prevotella nigrescens* (1)
BCH-261	8	26	----	*Prevotella copri* (1); *Prevotella bivia* (1); *Prevotella disiens* (1)	*Prevotella melaninogenica* (5); *Prevotella oris* (1); *Prevotella pallens* (2); *Prevotella nigrescens* (3); *Prevotella salivae* (1)
BCH-263	25	30	----	*Prevotella copri* (14)	*Prevotella melaninogenica* (8); *Prevotella shahii* (1); *Prevotella intermedia* (1); *Prevotella nanceiensis* (1)
BCH-313	5	31	----	*Prevotella copri* (3)	*Prevotella melaninogenica* (6); *Prevotella salivae* (2); *Prevotella oris* (1); *Prevotella pallens* (4); *Prevotella nigrescens* (1); *Prevotella intermedia* (1)
BCH-317	18	14	----	*Prevotella copri* (7); *Prevotella stercorea* (1)	*Prevotella pallens* (1); *Prevotella shahii* (1); *Prevotella oulorum* (1); *Prevotella nanceiensis* (1); *Prevotella aurantiaca* (1); *Prevotella melaninogenica* (1)
BCH-339	1	15	----	*Prevotella copri* (1)	*Prevotella melaninogenica* (5); *Prevotella pallens* (1); *Prevotella shahii* (1)
BCH-392	2	31	----	*Prevotella copri* (1)	*Prevotella melaninogenica* (9); *Prevotella shahii* (1); *Prevotella aurantiaca* (1); *Prevotella nanceiensis* (1); *Prevotella pallens* (2); *Prevotella salivae* (2);
BCH-405	18	48	----	*Prevotella copri* (7)	*Prevotella melaninogenica* (12); *Prevotella intermedia* (1); *Prevotella pallens* (3); *Prevotella shahii* (1); *Prevotella intermedia* (1); *Prevotella pallens* (3); *Prevotella salivae* (2)

The *Prevotella* had 828 16S rRNA gene sequences in 23 patients, among which 214 sequences from gut samples and 614 sequences from lung samples. We annotated 21 species, of which gut samples were annotated to 6 species and BALF samples to 24 species. No shared species were found. The sequences from paired fecal and BALF samples in LADC patients with distant phylogenetic distance and low sequence identity (< 97%) were not considered as shared species. The sequence identity of pairwise 16S rRNA gene sequences was analyzed using VSEARCH v2.15.0.

Table S8. Significantly different KEGG orthologous genes between the gut samples of patients with LADC and healthy controls in significantly different KEGG modules

KEGG ORTHOLOGY	Name	F-LADC: mean rel. freq. (%)	F-LADC: std. dev. (%)	F-HC: mean rel. freq. (%)	F-HC: std. dev. (%)	*P* values	95.0% lower CI	95.0% upper CI	KEGG Module
K01966	propionyl-CoA carboxylase [EC:6.4.1.3]	0.023465	0.017861	0.012878	0.010052	0.000912	0.004519	0.016654	Propanoyl CoA catabolism; Ethylmalonyl pathway
K01961	acetyl-CoA carboxylase [EC:6.4.1.2]	0.052121	0.02173	0.043406	0.018974	0.037353	0.000523	0.016908	Fatty acid biosynthesis
K00382	pyruvate dehydrogenase complex [EC:1.2.4.1 2.3.1.12 1.8.1.4]	0.048578	0.020847	0.039058	0.01675	0.015553	0.001859	0.017182	Leucine degradation; Pyruvate oxidation
K01745	histidine ammonia-lyase [EC:4.3.1.3]	0.034533	0.017744	0.026853	0.016359	0.027646	0.000865	0.014493	Histidine degradation
K01951	GMP synthase [EC:6.3.5.2]	0.087201	0.010508	0.08097	0.008504	0.001961	0.002361	0.010101	Guanine ribonucleotide biosynthesis
K00033	6-phosphogluconate dehydrogenase [EC:1.1.1.44 1.1.1.343]	0.024326	0.013907	0.018935	0.011962	0.043044	0.000173	0.010608	Pentose phosphate pathway
K00603	glutamate formiminotransferase [EC:2.1.2.5]	0.021237	0.012455	0.016133	0.01249	0.042778	0.00017	0.010039	Histidine degradation
K00036	glucose-6-phosphate 1-dehydrogenase [EC:1.1.1.49 1.1.1.363 1.1.1.388]	0.022359	0.011375	0.01745	0.011668	0.034659	0.000362	0.009457	Pentose phosphate pathway
K01057	6-phosphogluconolactonase [EC:3.1.1.31]	0.019245	0.010461	0.014347	0.010815	0.02263	0.000702	0.009093	Pentose phosphate pathway
K11381	2-oxoisovalerate dehydrogenase [EC:1.2.4.4]	0.009016	0.008943	0.004899	0.004759	0.008233	0.001105	0.007129	Leucine degradation
K01807	ribose 5-phosphate	0.012478	0.009045	0.008678	0.006094	0.019856	0.000621	0.00698	Pentose phosphat

	isomerase [EC:5.3.1.6]							e pathway
K14534	4-hydroxybutyryl-CoA dehydratase / vinylacetyl-CoA-Delta-isomerase [EC:4.2.1.120 5.3.3.3]	0.002948	0.001723	0.002232	0.001367	0.026528	8.58E-05	0.001348	Hydroxypropionate-hydroxybutylate cycle
K20862	5-amino-6-(5-phospho-D-ribitylamino)uracil phosphatase [EC:3.1.3.104]	0.003813	0.005955	0.006668	0.008236	0.041923	-0.0056	-0.00011	Riboflavin biosynthesis
K01681	aconitate hydratase [EC:4.2.1.3]	0.065962	0.008048	0.069282	0.008572	0.046556	-0.00659	-5.2E-05	Glyoxylate cycle
K00041	tagaturonate reductase [EC:1.1.1.58]	0.036087	0.006586	0.040167	0.007796	0.004778	-0.00688	-0.00128	D-Galacturonate degradation
K01685	altronate hydrolase [EC:4.2.1.7]	0.041854	0.009735	0.046497	0.010264	0.021289	-0.00858	-0.00071	D-Galacturonate degradation
K03154	thiS; sulfur carrier protein	0.035508	0.009259	0.041203	0.011863	0.006954	-0.0098	-0.00159	Thiamine biosynthesis
K00040	fructuronate reductase [EC:1.1.1.57]	0.024882	0.014868	0.032199	0.016792	0.021098	-0.01351	-0.00112	D-Glucuronate degradation
K00874	2-dehydro-3-deoxygluconokinase [EC:2.7.1.45]	0.086359	0.019497	0.098785	0.021128	0.0026	-0.0204	-0.00445	D-Galacturonate degradation; D-Glucuronate degradation

The statistical analysis was performed using the STAMP software. A two-sided Welch's *t* test was used to identify significantly different orthologous genes between the two groups, with $P < 0.05$ considered significant. rel. freq, relative frequency. std. dev, standard deviation. CI, confidence intervals.

Table S9. Virulence factors with the most differences between the gut samples of patients with LADC and healthy controls

Virulence factors	Categories in VFDB	Pathways in KEGG
acm	Adherence	----
gelE	Exoenzyme	Quorum sensing
clpP	Stress survival	Cell cycle - Caulobacter
rfaD	Immune modulation	Lipopolysaccharide biosynthesis
kdsB	Immune modulation	Lipopolysaccharide biosynthesis
lpxK	Immune modulation	Lipopolysaccharide biosynthesis
msbA	Immune modulation	ABC transporters
orfM	Immune modulation	----
rffG	Immune modulation	Streptomycin biosynthesis; Polyketide sugar unit biosynthesis; Acarbose and validamycin biosynthesis; O-Antigen nucleotide sugar biosynthesis; Biosynthesis of vancomycin group antibiotics
rfaF	Immune modulation	Lipopolysaccharide biosynthesis
lpxC	Immune modulation	Lipopolysaccharide biosynthesis
gmhA/*lpcA*	Immune modulation	Lipopolysaccharide biosynthesis
htrB	Immune modulation	Lipopolysaccharide biosynthesis
